# Chemotherapy versus personalized therapy for EGFR mutant lung adenocarcinoma resistance to EGFR-tyrosine kinase inhibitors: a retrospective dual-center study

**DOI:** 10.1186/s12890-024-02905-1

**Published:** 2024-02-24

**Authors:** Kan Jiang, Lin Wu, Xinlong Zheng, Yiquan Xu, Qian Miao, Xiaobin Zheng, Longfeng Zhang, Cheng Huang, Gen Lin

**Affiliations:** 1 Department of Thoracic Oncology, Clinical Oncology School of Fujian Medical University, Fujian Cancer Hospital, Fuzhou, China; 2 The Second Department of Thoracic Oncology, the Affiliated Cancer Hospital of Xiangya School of Medicine, Central South University, Hunan Cancer Hospital, Changsha, China; 3 Fujian Key Laboratory of Advanced Technology for Cancer Screening and Early Diagnosis, Fuzhou, China; 4 Interdisciplinary Institute for Medical Engineering, Fuzhou University, Fuzhou, China

**Keywords:** Non‐small cell lung cancer, EGFR-TKI resistance, Chemotherapy, Personalized therapy

## Abstract

**Background:**

Advanced lung adenocarcinoma patients often develop resistance to EGFR tyrosine kinase inhibitors (EGFR-TKIs), leaving uncertainties regarding subsequent treatment strategies. Although personalized therapy targeting individual acquired resistances (ARs) shows promise, its efficacy has not been systematically compared with platinum-containing doublet chemotherapy, a widely accepted treatment after EGFR-TKIs failure.

**Methods:**

A retrospective dual-center study was conducted involving patients with advanced lung adenocarcinoma and EGFR mutations who developed resistance to EGFR-TKIs between January 2017 and December 2022. Eligible patients were adults aged 18 years or older with an Eastern Cooperative Oncology Group score of 0–1, normal organ function, and no prior chemotherapy. Patients were divided into the chemotherapy group (CG) or personalized therapy group (PG) based on the treatment received after disease progression. The primary endpoints were progression-free survival (PFS) and objective response rate (ORR).

**Results:**

Of the 144 patients enrolled, there were 53 patients in the PG and 91 patients in the CG. The PG acquired resistance to EGFR-TKIs through the MET amplification (27, 50%) and small cell lung cancer transformation (16, 30%) and 18% of them reported multiple resistance mechanisms. The ORR of the PG was similar to that of the CG (34% vs. 33%, *P* = 1.0) and the PFS of the PG patients was not statistically different from that of their CG counterparts [4.2 months (95% CI: 3.6–4.8 months) vs. 5.3 months (95% CI: 4.6–6.0 months), *P* = 0.77].

**Conclusions:**

These findings suggest that the therapeutic efficacy of chemotherapy approximates to that of personalized therapy, which signifies that chemotherapy is still a reliable choice for patients who develop resistance to EGFR-TKIs and that further research is awaited to explore the benefit of personalized treatment.

## Introduction

Lung cancer is notorious for its high morbidity and mortality rate worldwide [1], in which non‐small cell lung cancer (NSCLC) accounts for 80%‐85% of the incidences [2]. In the NSCLC population, epidermal growth factor receptor (EGFR) gene is found in 10–20% of Caucasians and at least 50% of Asian NSCLC patients [3]. EGFR tyrosine kinase inhibitors (EGFR-TKIs) have dramatically improved survival outcomes and are the first-line treatment for patients with EGFR-mutants [4].

Unfortunately, patients undergoing tyrosine kinase inhibitor (TKI) treatment eventually develop acquired resistance (AR). The common ARs include: (i) secondary mutations to EGFR, such as T790M mutation, C797S mutation [5]; (ii) activation of alternative pathways, such as MET amplification [6], ERBB2 amplification [7]; (iii) activation of downstream targets, for instance, RAS-MAPK pathway signaling [8], PIK3CA mutations [9]; (iv) histologic transformation, for example, small-cell lung cancer (SCLC) transformation [10]; (v) others: fibroblast growth factor receptor (FGFR) amplification, cell cycle gene alterations [8]. Certain EGFR-mutant NSCLCs may harbor multiple mechanisms of EGFR-TKI resistance [9]. However, the potential mechanisms underlying the AR remains obscured in up to 50% of cases [11].

After the progression with EGFR-TKIs, different options are available, including chemotherapy and personalized therapy that is based on individual ARs. For example, the combined treatment with EGFR and MET-TKIs can inhibit the growth of EGFR-mutated NSCLC coupled with MET amplification [12]. The combination with 3rd and 1st or 2nd EGFR-TKIs is also a reasonable strategy against the AR of T790M-trans-C797S [13]. Except for the 3rd EGFR-TKIs, which is approved for the treatment of NSCLC patients with positive T790M mutation after developing AR to the first-line EGFR-TKIs [14], the efficacy of other personalized therapeutic strategies is largely compromised by small samples and absence of comparison with chemotherapy.

Here we conducted a retrospective multi-center study to explore the efficacy of chemotherapy and personalized therapy. We found that the efficacy of chemotherapy approximated to that of personalized therapy, which indicates that chemotherapy may still serve as a promising option for patients who develop resistance to EGFR-TKIs.

## Methods

### Patients

A retrospective study was conducted at two centers, Fujian Cancer Hospital and Hunan Cancer Hospital, to collect data of patients with advanced NSCLC from January 1, 2017, to December 31, 2022, in China. The inclusion criteria for eligible patients were as follows: (i) aged 18 years or older; (ii) histologically-confirmed lung adenocarcinoma; (iii) Stage IV according to American Joint Committee on Cancer (AJCC) (8th edition); (iv) an Eastern Cooperative Oncology Group (ECOG) score of 0–1 and normal organ functions; (v) EGFR sensitive mutations (deletion of exon 19 or the L858R mutation); (vi) patients who developed resistance after the treatment with the first- or second-generation EGFR-TKIs and were negative for T790M or patients who were positive for T790M after the treatment with the first- or second-generation EGFR-TKIs and developed resistance after the administration of the third-generation EGFR-TKI or those who developed resistance after initial treatment with the third-generation EGFR-TKI; (vii) available resistance mechanism confirmed by the next-generation sequencing (NGS) or Fluorescence in situ hybridization (FISH) after progression.

The eligible patients were divided into the Chemotherapy group (CG) or Personalized group (PG) according to the subsequent therapies after resistance to EGFR-TKIs. The CG patients received platinum-containing doublet chemotherapy, which was combined with or without antiangiogenic therapy and immunotherapy. Genetic testing post-EGFR TKI resistance was not deemed essential in this context. The therapies in the PG were prompted according to the genetic testing or histologic transformation. Data regarding the demographic information, tumor histology and molecular pathology, clinical treatments and outcomes were collected for the further analysis.

The NGS was performed in Geneplus-Beijing Institute or Fujian Cancer Hospital, which covered genomic regions of 1,021 cancer-related genes. The sample included the peripheral blood or frozen tissue [15]. The FISH test was conducted in Fujian Cancer Hospital.

### Response evaluation

The tumor response was assessed according to the Response Evaluation Criteria in Solid Tumors (RECIST version 1.1) in terms of partial response (PR), stable disease (SD), progressive disease (PD) or complete response (CR). The objective response rate (ORR) was defined as the percentage of patients with PR and CR. The disease control rate (DCR) was designated as the percentage of CR, PR and SD. The progression-free survival (PFS) was termed as the time from the initiation of treatment to disease progression or death from any cause. Treatment was considered censored if no evidence of progression was found at the last follow-up and time was recorded from start of treatment to the last follow-up.

### Statistical analysis

All data were analyzed using the SPSS 24.0 Software. Unadjusted PFS was estimated by the Kaplan–Meier product-limit method. The clinical and biological characteristics and ORR between two groups were analyzed by the chi-square test. The two-sided significance level was set at *P* < 0.05.

## Results

### The baseline characteristics were balanced between CG and PG

A total of 144 patients were enrolled, with a median age of 57 years, of whom, 86 patients reported a deletion of *EGFR* exon 19. The enrolled patients were further categorized into PG (53 patients) and CG (91patients). The baseline characteristics, including the incidence of EGFR exon 19 deletions and L858R mutations, were balanced between the two groups, with no significant statistical differences observed (all *P* > 0.05) (Table 1).

**Table 1:** Demographics and characteristics of patients

Characteristic	PG(*n* = 53)	CG(*n* = 91)	*P*
Age, years, mean (SD)	56.2(10.4)	56.2(8.0)	0.98
Gender			0.49
Male	20(37.7%)	40(44.0%)	
Female	33(62.3%)	51(56.0%)	
Smoking Status			0.43
Never	37(69.8%)	70(76.9%)	
Current/former	16(30.2%)	21(23.1%)	
ECOG Score			0.60
0	19(35.8%)	38(41.8%)	
1	34(64.2%)	53(58.2%)	
EGFR mutation			0.60
Exon 19 del	30(56.6%)	56(61.5%)	
Exon 21 L858R	23(43.4%)	35(38.5%)	
The therapy modes of TKIs			0.07
Only 1 or 2-generation TKIs	13(24.5%)	20(22.0%)	
Only 3rd-generation TKIs	16(30.2%)	14(15.4%)	
Sequential EGFR-TKIs	24(45.3%)	57(62.6%)	
Location of Metastases			
Brain	22(41.5%)	25(27.5%)	0.10
Liver	12(22.6%)	11(12.1%)	0.11
Bone	29(54.7%)	48(52.7%)	0.86

Values are expressed as median [interquartile range] or n (%). *PG* P*ersonalized* group, CG Chemotherapy group

### The efficacy was no significant difference between CG and PG

**Fig. 1:**
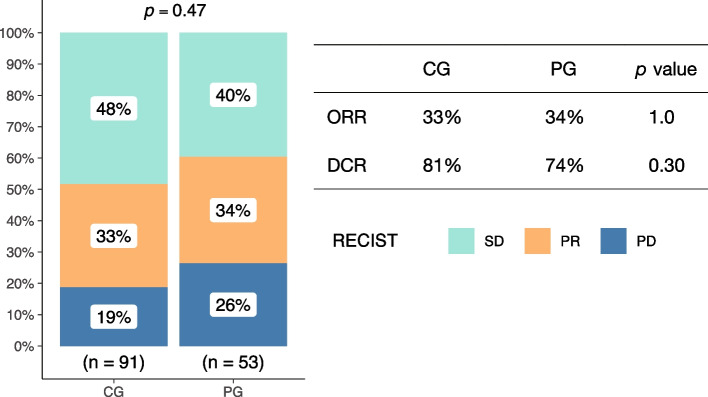
The ORR and DCR was no significant difference between CG and PG. *PG*, Personalized group; *CG*, Chemotherapy group; *ORR*, objective response rate; *DCR*, disease control rate

Among the 53 PG patients, PR was reported in 18 patients and SD in 21 patients. In the CG, PR was found in 30 patients and SD in 65 patients. No significant differences were observed in ORR (34.0% vs. 33.0%, *P* = 1.0) and DCR (73.6% vs. 81.3%, *P* = 0.30) between two groups (Fig. 1).

**Fig. 2:**
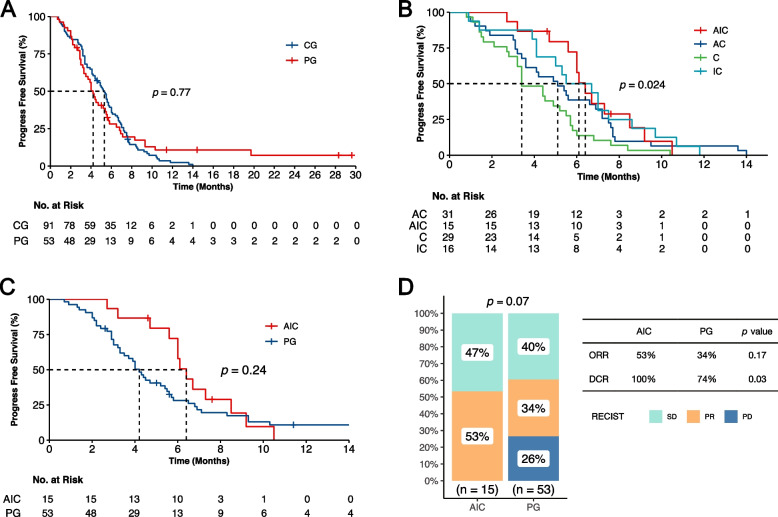
The PFS of different groups. **A** The PFS between CG and PG. **B** The PFS between four subgroups in CG. **C** Tumor response rates between AIC and PG. **D** The PFS between four subgroups in CG. *PG*, Personalized group; *CG*, Chemotherapy group; *C*, chemotherapy alone subgroup in CG; *AC*, anti-angiogenesis plus chemotherapy subgroup in CG; *IC*, immune checkpoint inhibitors plus chemotherapy subgroup in CG; *AIC*, a combination of immune checkpoint inhibitors, anti-angiogenesis and chemotherapy subgroup in CG. *ORR*, objective response rate; *DCR*, disease control rate

With the cutoff date set on March 15, 2023, the median follow-up time was 26.9 months. The disease progression was reported in 89 (97.8%) CG patients and 46 (86.8%) PG counterparts. The median PFS was 5.3 months (95% CI, 4.6–6.0 months) in CG, and 4.2 months (95% CI, 3.6–4.8 months) in PG (Fig. 2A), demonstrating no significant difference between two groups (HR, 0.95; *P* = 0.77).

In terms of EGFR 19 deletion, a cohort of 8 PG and 19 CG patients were PR, with ORR 26.7% (8/30), PFS 3.9 months (95% CI, 2.9–4.9 months) in PG and ORR 33.9% (19/56), PFS 4.9 months (95% CI, 3.4–6.4 months) in CG. With EGFR L858R, there were 10 PG and 11 CG patients were PR, with ORR 43.5% (10/23), PFS 4.5 months (95% CI, 2.4–6.6 months) in PG and ORR 31.4% (11/35), PFS 5.5 months (95% CI, 4.8–6.2 months) in CG. There were no statistic differences in ORR (*P* = 0.9) and PFS (*P* = 0.92) between PG and CG patients in terms of exons 19 and 21.

A cohort of 27 (50.9%) PG patients with MET amplification reported an ORR of 40.7% and a PFS of 4.2 m. Similar outcome was present in the SCLC transformation cohort (16, 30.2%), with an ORR of 37.5% and a PFS of 3.9 months (Table 2). Ten PG patients showed multiple resistance mechanisms, with an ORR of 20% and a PFS of 4.0 months.

**Table 2:** Resistant mechanism, individual therapy strategy and efficacy in PG

Rsistance mechanism	Drugs	Number	ORR	DCR	PFS (m)
MET amplification	MET plus EGFR-TKIs	27	40.7%	87.7%	4.2
SCLC transformation	Etoposide plus platinum or carboplatin	16	37.5%	75.0%	3.9
BRAF mutation	Dabrafenib, trametinib and EGFR-TKIs,	3	33.3%	33.3%	2.9
ERBB2 amplification	Trastuzumab, chemotherapy and EGFR-TKIs	4	0%	100%	-
RET fusion	LOXO-292 plus EGFR-TKIs	1	0%	0%	0.9
T790M-trans-C797S	the 1st and 3rd EGFR-TKIs	2	0%	50%	0.7

*PG* P*ersonalized* group, *ORR* Objective response rate, *DCR* Disease control rate, *PFS* Progression-free survival

The CG patients were further divided into the following subgroups on basis of the received treatment scheme: chemotherapy alone subgroup (C), anti-angiogenesis plus chemotherapy subgroup (AC), immune checkpoint inhibitors plus chemotherapy subgroup (IC) or a combination of immune checkpoint inhibitors, anti-angiogenesis and chemotherapy subgroup (AIC). The analysis revealed that the AIC subgroup with 15 (16.5%) patients reported significant difference in PFS (6.4 m vs. 3.4 m, *P* = 0.004) and ORR (53.3% vs. 20.7%, *P* = 0.022) when compared the C subgroup, but no statistical difference when in comparison with the IC and AC subgroups (Table 3**, **Fig. 2B).

**Table 3:** Efficacy of different subgroup in CG

	C(*n* = 29)	AC(*n* = 31)	IC(*n* = 16)	AIC(*n* = 15)
ORR	20.7%	38.7%	25%	53.3%
PFS(m,95% CI)	3.4(2.3–4.5)	5.1(3.5–6.6)	5.5(2.7–8.2)	6.4(5.7–7.1)

*CG* Chemotherapy group, *C* Chemotherapy alone subgroup, *AC* Anti-angiogenesis plus *chemotherapy subgroup*, IC Immune checkpoint inhibitors plus chemotherapy subgroup, *AIC* A combination of immune checkpoint inhibitors, anti-angiogenesis and chemotherapy subgroup, *ORR* Objective response rate, *PFS* Progression-free survival, *CI* Confidence interval

Continuing, we compared the AIC and PG groups. The analysis revealed a trend towards better outcomes in the AIC group, with a median PFS (6.4 m vs. 4.2 m, *P* = 0.24; Fig. 2C) and ORR (53.3% vs. 23.0%, *P* = 0.17; Fig. 2D) suggesting a potential advantage. Furthermore, the advantage in DCR reached statistical significance (100% vs. 73.6%, *P* = 0.032; Fig. 2D).

### The resistance mechanisms were complex after failure to EGFR-TKI

**Fig. 3:**
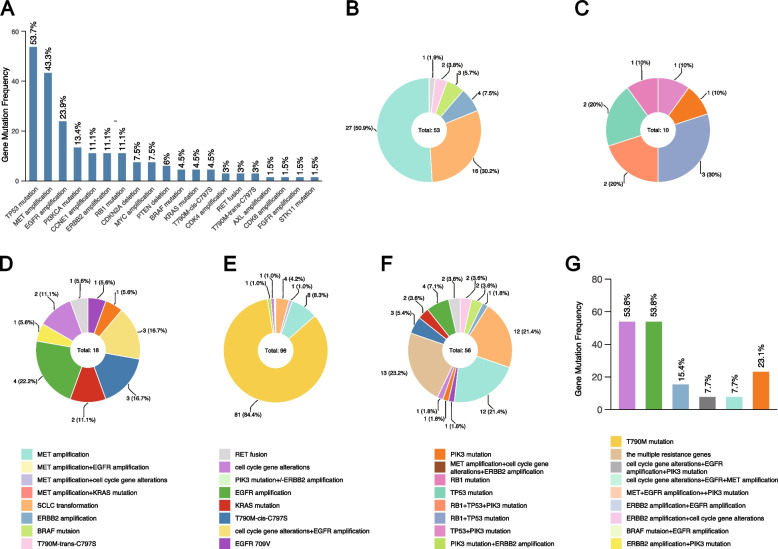
The resistance gene spectrum of EGFR mutation in NSCLC patients. **A** The gene change frequency after progression to EGFR-TKIs in NSCLC patients. **B** The main resistance mechanism in PG. **C** The gene change in SCLC transformation subgroup. **D** The main resistance mechanism in CG. **E** The resistance mechanism after failure to 1st or 2nd generation EGFR-TKIs. **F** The resistance mechanism to the 3rd generation EGFR-TKIs. **G** The co-occurring resistance genes in the research. *PG*, Personalized group; *CG*, Chemotherapy group

Among the 144 patients, 67 patients reported a definite resistance mechanism, with TP53 as the most frequent co-occurring mutation (36/67, 53.7%) and MET amplification as the main resistance mechanism (29/67, 43.2%) (Fig. 3A).

In the PG, 38 cases were detected by NGS and 9 were by FISH. The results showed that MET amplification (27/53, 50.9%) and SCLC (16/53, 30.2%) transformation were the most common AR types, which frequently received a personalized therapy (Fig. 3B). In the SCLC transformation samples, TP53, Rb1 and PIK3CA were the most common mutations (Fig. 3C). Of the 91 CG patients, 20 underwent NGS testing, with 18 reporting a detailed type of AR. Cell cycle gene alterations and EGFR amplification were the most common AR types (Fig. 3D).

The T790M (81 of 124 patients, 65.3%) was the most frequent AR for patients who were irresponsive to the 1st or 2nd generation EGFR-TKIs (Fig. 3E). The resistance mechanisms were complex and the multiple resistance genes more often appeared after the progression to the 3rd generation EGFR-TKIs (Fig. 3F). The alterations of cell cycle genes and EGFR amplification were the most common co-occurring resistance genes (Fig. 3G).

## Discussion

For patients with disease progression after EGFR-TKI failure, the optimal treatment strategy remains controversial and no consensus has been reached. Our retrospective study found that the therapeutic efficacy of chemotherapy was comparable to that of personalized therapy. Interestingly, we found that when chemotherapy was supplemented with immune checkpoint inhibitors (ICIs) and anti-angiogenic drugs, there was a trend towards providing additional clinical benefits compared to personalized therapy. This highlights the enduring clinical significance of chemotherapy for patients experiencing relapse after prior EGFR-TKI therapy. Moreover, in some cases, combination therapy based on chemotherapy may provide incremental benefits. This emphasizes that chemotherapy is a promising option for treating patients with EGFR-TKI resistance.

PFS with traditional chemotherapy offers a length of 4.4–5.4 months for previously EGFR-TKI treated patients [16]. The data from clinical trials have not shown substantial survival benefits of single-agent ICI [17]. The combination therapy of chemotherapy and ICI or chemotherapy with anti-angiogenesis reported an efficacy with an ORR of 30–60% and a PFS of 5.0–7.0 m [18–20]. ICIs combined with chemotherapy and anti‐angiogenic drugs showed excellent benefits for patients receiving prior EGFR-TKI treatment, with an ORR of 73.5% and a PFS of 10.2 m in the IMpower150 subgroup, and an ORR of 43.9% and a PFS of 6.9 m in the ORIENT-31 trail [21–23]. In our study, the efficacy of the combined modality was superior to the chemotherapy alone, particularly in the “quad” model. The patients with EGFR mutations reported a high Treg infiltration, reduced CD8 + T-cell number and decreased tumor mutation burden (TMB) [24–26], which induced a poor clinical efficacy of ICI. EGFR-TKIs can remodel the tumor microenvironment (TME) by increasing CD8^+^ T cell infiltration and the presentation of MHC class I and II molecules, reducing the infiltration and function of Tregs [27]. So immunotherapy is applied after EGFR-TKIs failure. However, the efficacy and responsiveness of ICI monotherapy are far from satisfactory. Anti‐angiogenic drugs can reduce hypoxia, increase the delivery and efficacy of cytotoxic agents, and reduce immunosuppression through preventing angiogenesis and normalizing the tumor vasculature [28]. Consistent with the previous study, the current study revealed a satisfactory outcome when anti-angiogenics was combined with chemotherapy and immunotherapy. It would be beneficial to conduct further large-scale and prospective studies to confirm these results.

To date, many efforts have been invested in the search for effective treatment strategies to overcome EGFR-TKI resistance according genetic testing and histologic transformation. In case of EGFR C797S mutation, the follow-up treatment depends on the allelic relationship with T790M: T790M-trans-C797S is sensitive to the combination of first and third-generation of EGFR-TKIs [29], and a combination of brigatinb with cetuximab can achieve a favorable outcome with a PFS of 14 months and an ORR of 60%, in patients with T790M-cis-C797S [30]. The concomitant treatment with Trastuzumab and EGFR-TKIs has been demonstrated to possibly overcome the resistance to EGFR-TKIs as result of ERBB2 amplification [31]. SCLC transformation is responsive to platinum etoposide regimens with a PFS of 3.0–4.0 m [10, 32]. A retrospective trial found it is invalid to ICIs [10]. But another retrospective trial discovered that immunochemotherapy significantly prolonged the OS than chemotherapy. Positive PD-L1 status was associated with PFS benefit. And the expression of SFTPA1 in RNA sequencing predicted the durable clinical benefit. Large sample and prospective studies were needed to explore the efficacy of ICIs in SCLC transformation patients [33]. In our study, the patients reported a PFS of 4.2 m and an ORR of 34% in the PG, which is no better than the data in the CG and the data of previous studies, demonstrating that chemotherapy is an acceptable choice after the EGFR-TKIs failure. The above-mentioned findings also suggest that the evidence from personalized treatment is insufficient, for most of the above data are derived from retrospective research, preclinical studies, or small sample trials.

MET amplification has been implicated as one of the bypass resistance mechanisms to EGFR-TKI therapy. Numerous case reports and a growing number of clinical studies have documented the efficacy of the combinatorial regimen of EGFR-TKI and MET-TKI in simultaneously inhibiting both EGFR and MET signaling pathways to overcome EGFR-TKI resistance [34]. The combination of Tepotinib and Gefitinib has reported an amazing efficacy, with an ORR of 66.7% and a PFS of 16.6 m in the INSIGHT study [12]. Osimertinib plus savolitinib demonstrates a strong anti-tumor activity, with an ORR of 52% and a median duration of response (DOR) of 7.1 months in the TATTON Phase Ib expansion cohort [35]. In some trials and the real-world study, however, other MET inhibitors combined with EGFR-TKIs report a PFS of only 5-6 m [34, 36–38]. Amivantamab (EGFR-MET bispecific antibody) with lazertinib has shown anti-tumor activity with ORR 36% and PFS 4.9 m in patients of disease progression upon EGFR-TKI [39]. But emibetuzumab (monoclonal bivalent MET antibody) plus erlotinib could not reverse the AR to EGFR-TKI [40]. In our research, the PFS was 4.2 m and ORR was 40.7% in patients with MET amplification in the PG. Therefore, the “quad” model chemotherapy may be a favorable choice to these patients and further studies are awaited to explore the beneficiary group of a personalized therapy.

The analysis of circulating tumor DNA from NSCLC patients reveals that 46% of patients treated with EGFR-TKIs may have multiple resistance mechanisms [41]. Alterations to cell cycle gene and the PI3K pathway were the most common co-occurring resistance mechanism [42]. The multiple resistance mechanisms pose a challenge to personalized therapy. Our study found that 13 patients had the multiple resistance mechanisms; cell cycle gene alterations and EGFR amplification were the most common co-occurring resistance mechanisms; 10 PG patients displayed multiple resistance mechanisms, with an ORR of 20% and a PFS of 4.0 m, which indicated no impact on the efficacy of the therapy.

Some limitations remain in this retrospective study. First, the data of overall survival and adverse events were not available, which may affect the benefit elucidation between the CG and PG. Second, only a small number of patients received ICI plus chemotherapy with or without anti‐angiogenic drugs, which might limit the interpretation to determine the optimal therapeutic strategy. Third, the data regarding PD-L1 expression were insufficient to determine whether PD-L1 expression was balanced across groups or to analyze the correlation between PD-L1 expression and ICI efficacy. There is not ongoing study to compare OS between two therapy modes, a prospective study in these patients can be conducted.

## Conclusion

This retrospective study demonstrates that chemotherapy, especially combined with antiangiogenic therapy and immunotherapy, may serve as the standard treatment strategy for patients who experience disease progression after EGFR-TKIs failure.

## Data Availability

The datasets used and/or analyzed during the current study available from the corresponding author on reasonable request.

## Abbreviations

NSCLC: Non-small cell lung cancer
EGFR: Epidermal growth factor receptor
EGFR-TKIs: EGFR tyrosine kinase inhibitors
TKI: Tyrosine kinase inhibitor
AR: Acquired resistance
SCLC: Small-cell lung cancer
FGFR: Fibroblast growth factor receptor
AJCC: American Joint Committee on Cancer
ECOG: Eastern Cooperative Oncology Group
NGS: Next-generation sequencing
FISH: Fluorescence in situ hybridization
CG: Chemotherapy group
PG: Personalized group
RECIST: Response Evaluation Criteria in Solid Tumors
PR: Partial response
SD: Stable disease
PD: Progressive disease
CR: Complete response
ORR: Objective response rate
DCR: Disease control rate
PFS: Progression-free survival
ICI: Immune checkpoint inhibitors
TME: Tumor microenvironment
TMB: Tumor mutation burden
DOR: Duration of response
